# Effect of subject‐specific head morphometry on specific absorption rate estimates in parallel‐transmit MRI at 7 T

**DOI:** 10.1002/mrm.29589

**Published:** 2023-01-19

**Authors:** Hongbae Jeong, Jesper Andersson, Aaron Hess, Peter Jezzard

**Affiliations:** ^1^ Wellcome Centre for Integrative Neuroimaging, FMRIB Division, Nuffield Department of Clinical Neurosciences University of Oxford Oxford UK; ^2^ Athinoula A. Martinos Center for Biomedical Imaging, Department of Radiology Massachusetts General Hospital Boston Massachusetts USA; ^3^ Centre for Clinical Magnetic Resonance Research, Department of Cardiovascular Medicine University of Oxford Oxford UK; ^4^ British Heart Foundation Centre for Research Excellence Oxford UK

**Keywords:** electromagnetic body models, MRI safety, nonlinear registration, parallel transmit, RF transmit, SAR

## Abstract

**Purpose:**

To assess the accuracy of morphing an established reference electromagnetic head model to a subject‐specific morphometry for the estimation of specific absorption rate (SAR) in 7T parallel‐transmit (pTx) MRI.

**Methods:**

Synthetic T_1_‐weighted MR images were created from three high‐resolution open‐source electromagnetic head voxel models. The accuracy of morphing a “reference” (multimodal image‐based detailed anatomical [MIDA]) electromagnetic model into a different subject's native space (Duke and Ella) was compared. Both linear and nonlinear registration methods were evaluated. Maximum 10‐g averaged SAR was estimated for circularly polarized mode and for 5000 random RF shim sets in an eight‐channel transmit head coil, and comparison made between the morphed MIDA electromagnetic models and the native Duke and Ella electromagnetic models, respectively.

**Results:**

The averaged error in maximum 10‐g averaged SAR estimation across pTx MRI shim sets between the MIDA and the Duke target model was reduced from 17.5% with only rigid‐body registration, to 11.8% when affine linear registration was used, and further reduced to 10.7% when nonlinear registration was used. The corresponding figures for the Ella model were 16.7%, 11.2%, and 10.1%.

**Conclusion:**

We found that morphometry accounts for up to half of the subject‐specific differences in pTx SAR. Both linear and nonlinear morphing of an electromagnetic model into a target subject improved SAR agreement by better matching head size, morphometry, and position. However, differences remained, likely arising from details in tissue composition estimation. Thus, the uncertainty of the head morphometry and tissue composition may need to be considered separately to achieve personalized SAR estimation.

## Abstract 2

Click here for author‐reader discussions

## 1 INTRODUCTION

Computational modeling and simulation have been used extensively to aid the design, optimization, efficacy, and safety testing of medical devices.^1^ The scope of computational modeling includes, but is not limited to, optics, thermodynamics, fluid dynamics, ultrasound, mechanics, and electromagnetics (EM). Computational modeling in MRI has been used for various purposes, such as estimation of signal intensity and SNR at different main magnetic field strengths,^2^ optimization of RF transmit and B_0_ shimming fields, and for assessment of RF and gradient field interactions with the human body.^3^, ^4^, ^5^, ^6^, ^7^, ^8^ To aid this, virtual human models have been generated and are valuable tools in estimating complex RF field interactions on biological tissues. A realistic anatomical representation of virtual human models plays an important role in accurately estimating the effects of RF field exposure on human tissue. One of the well‐known image‐based whole‐body models is the visible photographic man (VIP‐MAN), segmented from cadaver images of a 39‐year‐old male as part of the Visible Human Project.^9^ The Virtual Population was introduced more recently by the IT'IS Foundation (Zürich, Switzerland) with ages ranging between 8 weeks and 80 years, segmented from medical images.^10^ There is also the multimodal image‐based detailed anatomical (MIDA) head and neck model with 115 structures, including nerve tracts and deep brain structures, which is considered one of the most accurate image‐based head and neck models.^11^

Parallel‐transmit (pTx) MRI is an invaluable technique to mitigate B_1_^+^ field inhomogeneity in high‐field MRI such as 7 T. Although higher SNR can be achieved at higher magnetic field strength, the B_1_^+^ field patterns are complex due to the smaller wavelength of the EM fields relative to the size of the head or body. The safety of RF exposure on the human body is usually controlled by a precalculated specific absorption rate (SAR) based on a standard virtual model as an indirect method to estimate the SAR experienced by a given subject. Therefore, a safety margin must be applied to the generic EM calculation to account for variations in head morphometry across the population. The use of a subject‐specific voxel model could reduce the required safety margin that must be applied to account for uncertainties coming from patient head position and morphometry inside the transmit coil, and thus could lead to shorter scan times or better optimized RF pulses. According to the International Electrotechnical Commission (IEC) 60601‐2‐33 guideline,^12^ the RF SAR safety limit for the case of head imaging is 3.2 W/kg average in the head for a volume transmit coil, and 10 W/kg in any 10‐g mass–averaged tissue for a local transmit coil in normal operation mode.

Different approaches have been attempted to estimate a truly patient‐specific SAR to run the scanner closer to the true SAR limit. One example is electrical properties tomography, which can be used to estimate the personalized dielectric properties of the subject.^13^ However, it requires reliable calibration, validation using a phantom to characterize accurate conductivity and permittivity of the different types of brain tissue compartments in the subject, as well as several MRI scans to calculate and map the dielectric properties of the head.

Another approach is to use direct segmentation of structural images to account for the personalized tissue composition of a particular subject in the scanner.^14^, ^15^, ^16^ However, accurate segmentation of the subject's head is challenging due to a lack of signal in certain tissues (e.g., skull). Furthermore, segmentation of the head to achieve a subject‐specific voxel model also requires scans using multiple sequences, thus leading to scan overhead to acquire high‐resolution multimodality images.^10^, ^11^ An automated segmentation tool can be used that can generate a subject‐specific model with minimum anatomical knowledge.^17^, ^18^ However, manual refinements are often required to reduce the error that might occur from automated segmentation.^19^ In addition, the number of available tissue compartments is limited depending on the tools, and on the number of multicontrast MR images used. An alternative strategy that has been suggested is to use a subject‐specific registration to a “standard” EM template to assess the SAR in a 7T single‐channel volume head transmit coil.^20^ That study demonstrated the effect of head position and morphometry on SAR prediction and demonstrated good agreement for single‐transmit systems after morphing a template into a subject‐specific model. It remains unclear whether these results translate to pTx applications.

In this study, we conducted numerical simulations to investigate the effects of head morphometry on SAR in pTx 7T head MRI. Rigid body, affine linear (LIN), and nonlinear (NONLIN) image registration methods were used to register a “reference” EM model to a different test subject using anatomically accurate male and female whole‐body models. We hypothesized that accounting for the test subject's head position and head morphometry could mitigate uncertainties in SAR estimation. The effect of head morphometry in maximum 10‐g mass–averaged SAR (10gSAR) estimation was compared using 5000 random RF shim sets when using both LIN and NONLIN registration. Uncertainty was analyzed using two versions of one of the models that share identical head morphometry.

## 2 METHODS

### 2.1 EM models

Existing publicly available EM models were used to provide both the “reference” head model, as well as the “test” subjects. For the reference model, the MIDA (female, 29 years old) head model^11^ was used. For the test subjects, the Duke version 3.0 (male, 34 years old) whole‐body model and the Ella version 3.0 (female, 26 years old) whole‐body model^10^ were used. For the head registration process, the Duke and Ella whole‐body voxel models were cut at the level of the T5 vertebrae to match the size and level of the MIDA model. To mirror the proposed framework of morphing MIDA into other subject‐specific reference frames, simulated T_1_‐weighted data sets of the head were created from the MIDA, Duke, and Ella EM models, respectively. The simulated MRI tissue intensities were taken from a T_1_‐weighted image acquired separately in a volunteer subject under an institutionally approved technical development protocol using a 3T Siemens Prisma MRI scanner (Siemens Healthineers, Erlangen, Germany) using a 64‐channel head and neck coil with a T_1_ MPRAGE pulse sequence (TR/TE = 2380/3.96 ms, TI = 1200 ms, flip angle = 8°). T_1_‐weighted image intensities were imported into the MIDA, Duke, and Ella voxel models. To avoid EM discontinuities at the neck, the shoulders and trunk from the Electronics & Telecommunications Research Institute (ETRI) vk‐male and female models^21^ were added to the reference MIDA EM head model and to the warped MIDA EM head models. ETRI whole‐body models are open‐source models that consist of 102 compartments with 3‐mm voxel size in .stl format.

### 2.2 LIN and NONLIN registration of the voxel model

**FIGURE 1:**
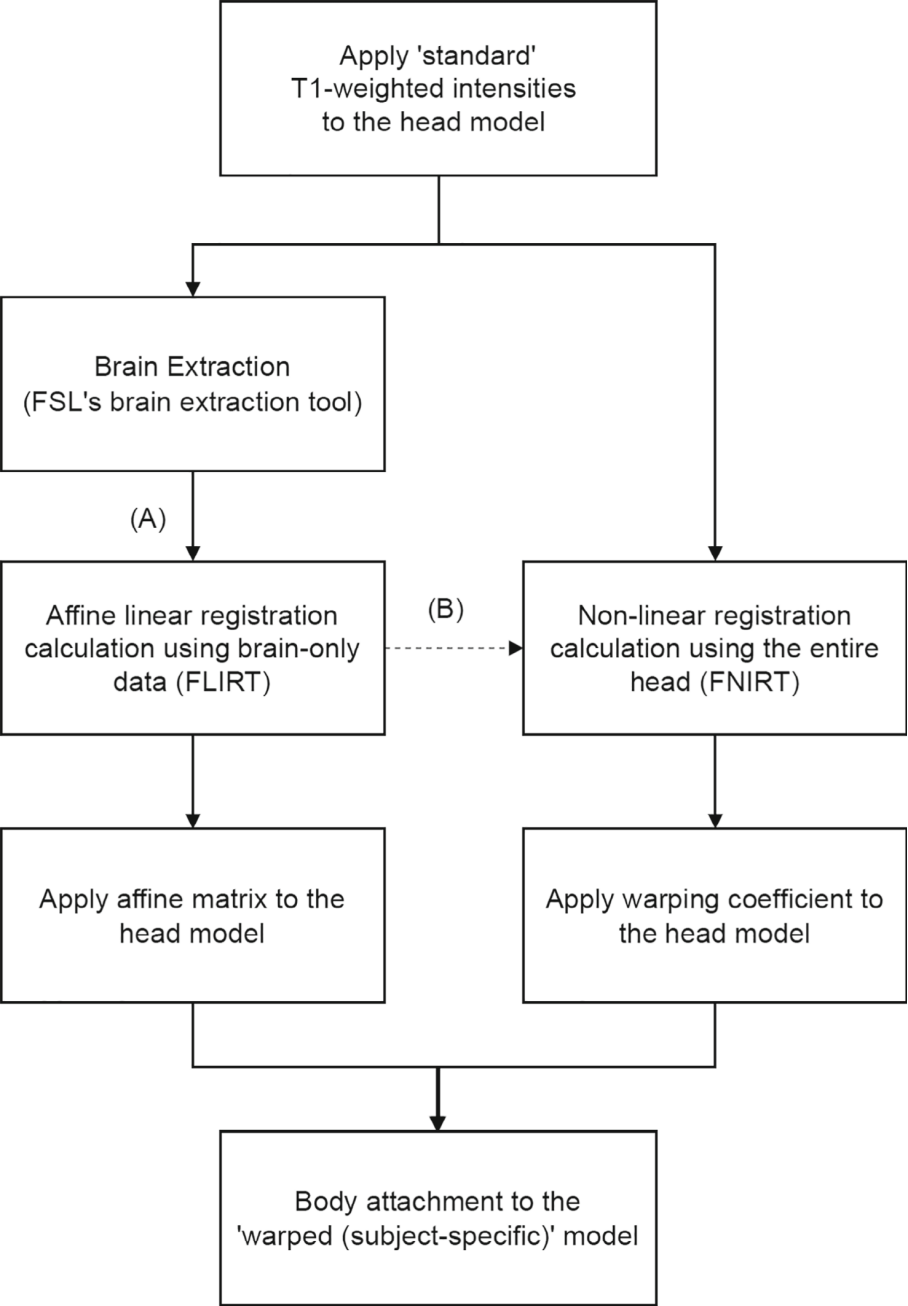
Flow chart of the subject‐specific model generation. A, Linear registration process via the extracted brain. B, Nonlinear registration of the entire head using the affine matrix calculated from brain‐only information as a starting point

A flow chart detailing the method of affine LIN or NONLIN registration of the high‐resolution MIDA voxel model into a different personalized voxel model is shown in Figure 1. Note that, additionally, simulations without registration (i.e., head centered inside the coil) were used as the comparison “no registration” procedure. The FLIRT (affine LIN) and FNIRT (NONLIN) registration tools from the FMRIB Software Library (FSL) were used to register the voxel models.^22^ The MIDA model was down‐sampled from (0.5 mm)^3^ to (2 mm)^3^ to reduce the registration processing time and memory requirements. Using this approach, it is possible to perform EM simulations of the MIDA model with full resolution morphed into Duke or Ella space, and to compare the SAR predictions with SAR simulations performed on the native Duke and Ella models themselves. The registration steps used were as follows:

- LIN registration: 12‐degrees‐of‐freedom affine matrices were calculated to map the Duke and Ella models into the space of the MIDA model using FLIRT (FMRIB's Linear image registration tool).^23^ Brain‐extracted versions (using Brain Extraction Tool, FSL's brain extraction tool) of the images were used to drive the registrations.
- NONLIN registration: A NONLIN registration was performed using FNIRT (FMRIB's Nonlinear image registration tool). The affine transform described above was used for initialization, and whole‐head data were used to drive the registration.
- A warp field (or affine matrix in the case of LIN registration) was then applied to the whole‐head MIDA model (in .stl format) to warp it into Duke or Ella space. For code details see Figure S1.
- To avoid boundary artifacts in the subsequent RF modeling, each registered MIDA EM head model was attached to the male or female ETRI whole‐body model, as appropriate, using Autodesk Meshmixer (Autodesk, San Rafael, CA, USA) (see Figure S2).

### 2.3 EM simulations

**FIGURE 2:**
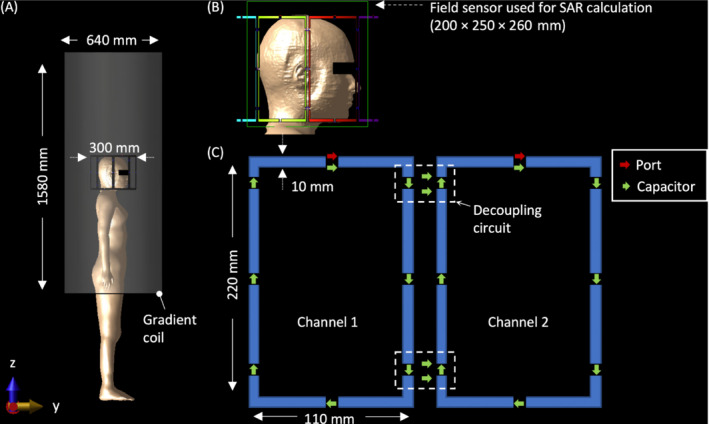
Numerical simulation setup. A, Multimodal image‐based detailed anatomical (MIDA) head model attached to the Electronics & Telecommunications Research Institute (ETRI) female whole‐body model positioned at the center of the RF coil with a gradient coil as a shield. B, Field sensor region where specific absorption rate (SAR) calculation is made. C, Coil element dimensions, position of the lumped elements, and the position of the decoupling circuit

All EM simulations were performed using *Sim4Life* (ZMT, Zürich, Switzerland). The coil model was based on an eight‐channel transmit/receive array coil (Affinity Imaging, Jülich, Germany), which has a 300‐mm diameter (lateral and longitudinal length of each element: 110 and 220 mm; 10‐mm strip width). Two capacitors were placed at the longitudinal side of each element to account for the decoupling circuit between neighboring elements (Figure 2). The coil response was tuned such that *S*_ii_ = −19.7 ± 6.7 dB and *S*_ij_ = −14.3 ± 4.8 dB at 297.2 MHz using Optenni Lab,^24^ with the MIDA model loading at the center of the coil. For all other models, *S*_ii_ < −9.5 dB and *S*_ij_ < −9.0 dB (Figure S7). Each head model was inserted inside the coil model for EM simulation. An SC72 gradient coil (Siemens Healthineers, Erlangen, Germany) was included in the model (length = 158 cm, diameter = 64 cm), modeled as a perfect electric conductor RF shield. The human voxel models were simulated at 297.2 MHz to assess the SAR. The Duke, Ella,^10^ and MIDA voxel models^11^ were positioned at the center of the coil, along with the MIDA model warped into Duke space (denoted as MIKE) and the MIDA model warped into Ella space (denoted as MILLA). Both LIN and NONLIN registrations of MIKE (MIKE_LIN_ and MIKE_NONLIN_) and MILLA (MILLA_LIN_ and MILLA_NONLIN_) were considered. Unless otherwise stated, all fields were normalized to 1 W conducted power per channel.

### 2.4 Evaluation of the registration

The Dice similarity coefficient (DSC) was used to assess the performance of the registration. The substructures of the tissue compartments in the MIDA model that shared dielectric properties with gray matter (e.g., pallidum, nucleus accumbens, substantia nigra, amygdala, mammillary body, caudate, putamen, hippocampus, hypothalamus, thalamus) were assigned the segmentation label of gray matter. The following equation was used to calculate the DSC:

(1) $$\mathrm{DSC}=\frac{2\left|X⋂Y\right|}{\left|X\right|+\left|Y\right|}$$

where *X* is the relevant labeled region in the target voxel model (i.e., Duke or Ella), and *Y* is the labeled region in the voxel model to be compared (i.e., MIDA, MIKE, or MILLA). The difference in electrical conductivity and tissue densities, which are key components when calculating SAR, were calculated using the tissue compartment characteristics of each model. Furthermore, the difference in |E‐field|^2^ was assessed between voxel models. (Density maps were also used to estimate the overall mass of the head model before and after warping.)

### 2.5 Circularly polarized mode and pTx mode analysis

SAR results were averaged over 10 g–mass subvolumes using the IEEE/IEC 62704‐1 standard,^25^ with the region of the field sensor as shown in Figure 2B when calculating the head‐averaged SAR (SAR_head_) and maximum 10gSAR. When simulating circularly polarized (CP) mode, the B_1_^+^ field, SAR, and 10gSAR were normalized with normalization factor (V) to fields that produced 2 μT at the coil center.^26^ Total absorbed power in the head (W) was computed as the integral of electric loss density (W/m^3^) in the head. For the pTx safety assessment, eight separate simulations were performed by adding 50‐Ω resistances to the ports corresponding to the nonactivated channels. For local SAR calculation in the case of the RF shim sets, 5000 sets with random phase (0‐2π) and random relative magnitude (0–1) were generated and applied on eight separate simulation results via a Python script, and the data were normalized to generate a total conducted power of 5 W when excited by all eight sources (see Figure S3 for the Python code).

### 2.6 Uncertainty analysis

To assess the effects of uncertainties in the maximum 10gSAR estimation in pTx MRI that are unrelated to head morphometry (e.g., errors created in tissue segmentation), the Duke version 1.0 (IT'IS Foundation) model was used with the same 5000 random RF shim sets as applied to the Duke version 3.0 model. Duke version 1.0 is the original virtual family model introduced in 2010, segmented and developed on the basis of medical images of the same subject as used for Duke version 3.0. As such, it has the same underlying morphometry, but was segmented separately. The data from Duke version 1.0 comprised a 3D volume, with 0.5 × 0.5 × 0.5 mm resolution, and surface meshes were generated using the mesh generation tool in Sim4Life (see Figure S4 for more details).

### 2.7 Effects of virtual observation point compression

The virtual observation point (VOP) compression algorithm^27^ was used to assess the maximum 10gSAR at maximum 5% overestimation for 5000 random B_1_^+^ shim sets (Figure S5). VOP compression was done via an enhanced VOP compression algorithm developed by Orzada et al.,^28^ resulting in 19.7 million 8 × 8 matrices being compressed to as few as 79 matrices (numbers of 8 × 8 matrices after compression at maximum 5% overestimation were Duke = 79, MIKE_LIN_ = 95, MIKE_NONLIN_ = 124, MIDA = 158, Ella = 280, MILLA_LIN_ = 151, and MILLA_NONLIN_ = 183).

## 3 RESULTS

### 3.1 Evaluation of LIN and NONLIN warping between the reference and test subject models

Table 1 provides the results of the DSC analysis of eight tissue classifications when using LIN and NONLIN registrations for the cases of Duke versus MIDA, Duke versus MIKE, Ella versus MIDA, and Ella versus MILLA. The DSC values were increased over simple rigid‐body registration when affine LIN registration was performed between MIDA and the subject space, and further improved when using the NONLIN registration. Poor native registration of the cerebellum between MIDA and the two “test” subject models showed the highest improvement of DSC with NONLIN registration (Duke = 0.93, Ella = 0.95), with muscle, CSF, and fat showing the lowest improvement in DSC, with results that remained below 0.60, but still considerably improved over a simple rigid‐body registration.

**TABLE 1:** Dice similarity coefficient analysis comparing MIDA versus Duke and Ella, versus comparing MIDA‐warped‐into‐Duke (MIKE_LIN_ and MIKE_NONLIN_) and MIDA‐warped‐into‐Ella (MILLA_LIN_ and MILLA_NONLIN_)

Tissue types	MIDA vs. Duke	MIKE_LIN_ vs. Duke	MIKE_NONLIN_ vs. Duke	MIDA vs. Ella	MILLA_LIN_ vs. Ella	MILLA_NONLIN_ vs. Ella
Muscle	0.59	0.55	0.59	0.45	0.55	0.56
Gray matter^a^	0.37	0.54	0.61	0.37	0.54	0.67
Skull	0.18	0.54	0.59	0.27	0.42	0.68
White matter	0.50	0.64	0.70	0.43	0.62	0.69
CSF	0.16	0.39	0.54	0.14	0.30	0.53
Cerebellum	0.81	0.83	0.93	0.68	0.87	0.95
Fat^b^	0.20	0.38	0.56	0.19	0.35	0.51
Skin	0.09	0.20	0.60	0.11	0.26	0.64

^a^ Gray matter, amygdala, caudate nucleus, globus pallidus, mammillary body, nucleus accumbens, putamen, and substantia nigra were grouped as gray matter.

^b^ Fat, tendon, and subcutaneous adipose tissue were grouped as fat.

### 3.2 Conductivity, density, and |E‐field|^2^ difference maps

**FIGURE 3:**
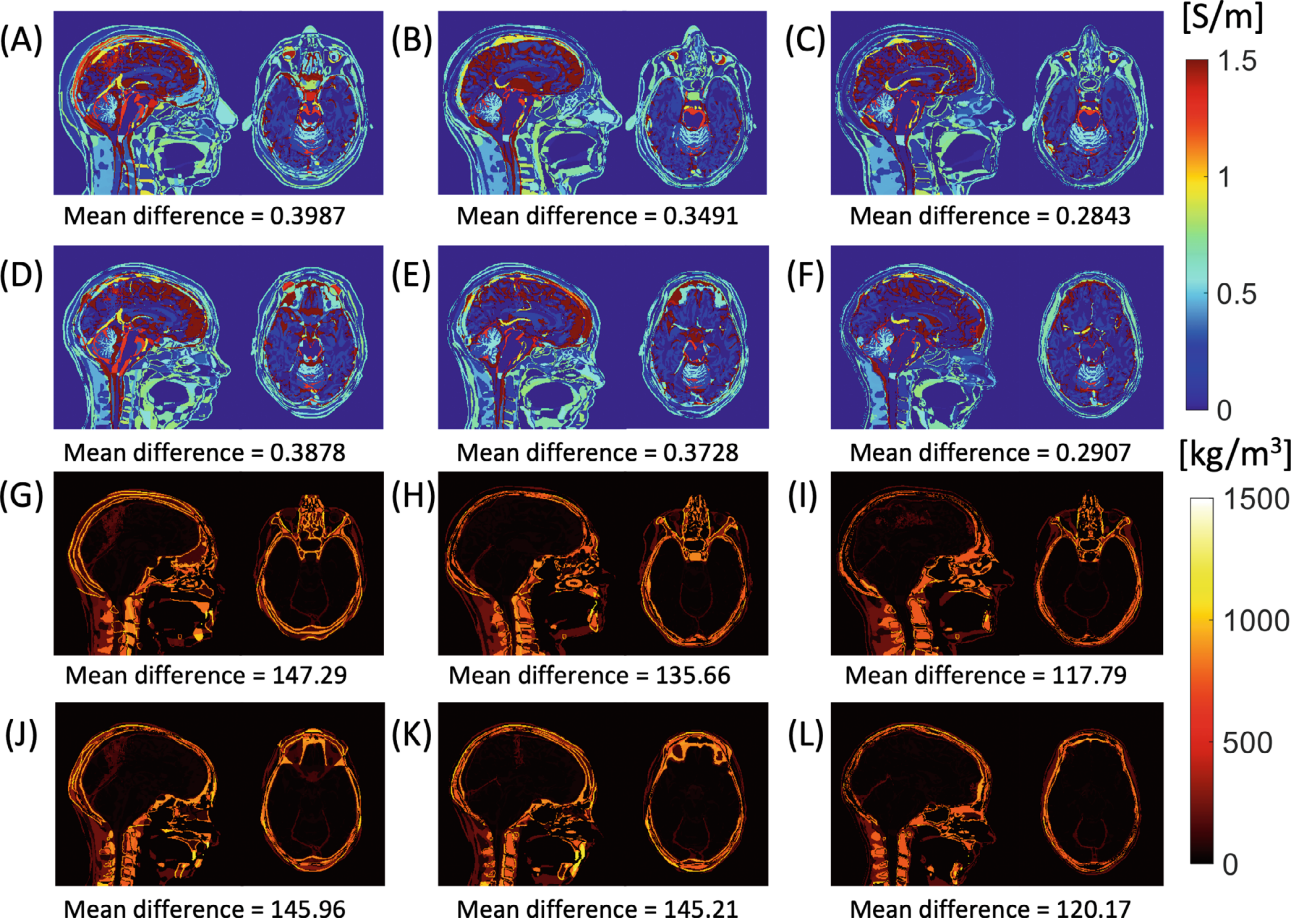
The fidelity of voxel electromagnetic (EM) properties. Absolute conductivity differences of MIDA–Duke (A); MIKE_LIN_–Duke (B); MIKE_NONLIN_–Duke (C), MIDA–Ella (D), MILLA_LIN_–Ella (E); MILLA_NONLIN_–Ella (F); and absolute density differences of MIDA–Duke (G); MIKE_LIN_–Duke (H); MIKE_NONLIN_–Duke (I); MIDA–Ella (J), MILLA_LIN_–Ella (K), and MILA_NONLIN_–Ella (L)

The difference in conductivity and density between Duke versus MIDA, and Ella versus MIDA, at the coil center and using affine and NONLIN registration is shown in Figure 3. The mean difference in conductivity between MIDA and Duke was reduced from 0.40 to 0.35 S/m (i.e., 12.4% decrease in mean difference) when the MIDA model was aligned with affine LIN registration into Duke space. NONLIN registration reduced the conductivity difference further to 0.28 S/m (i.e., 28.7% decrease in mean difference). The mean difference in density in the head was also reduced when MIDA was aligned with affine LIN registration into Duke space (from 147.29 to 135.66 kg/m^3^, yielding a 7.9% decrease in mean density difference). NONLIN registration further reduced this to 117.8 kg/m^3^ (20.0% decrease in mean density difference). A similar trend was found in mean density differences between MIDA and Ella (i.e., 3.9% decrease in mean conductivity difference for affine LIN registration (25.0% for the NONLIN registration), and 0.5% decrease in mean density difference for affine LIN registration (17.7% for NONLIN registration).

**FIGURE 4:**
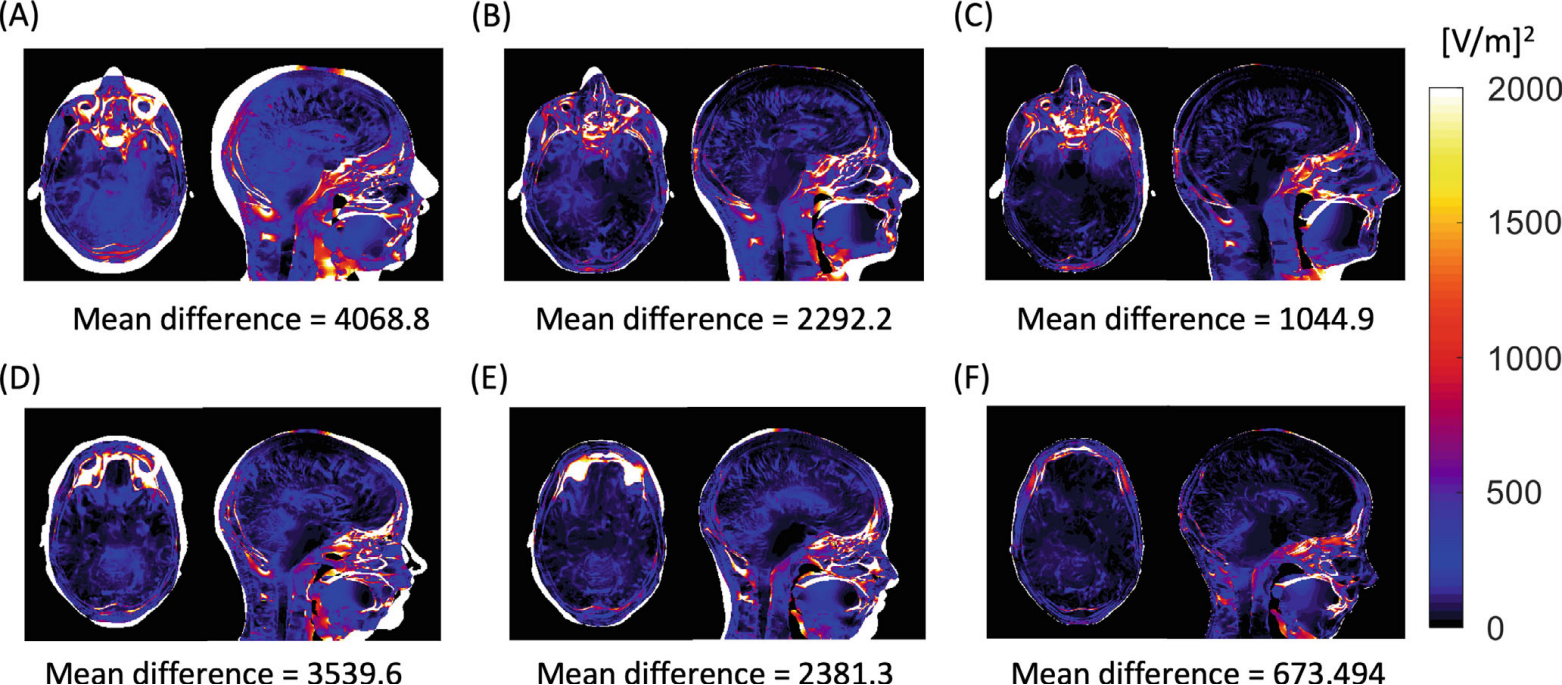
The fidelity of voxel EM properties. Mean squared differences in |E‐field|^2^ for Duke versus MIDA, MIKE_LIN_ and MIKE_NONLI_, and for Ella versus MIDA, MILLA_LIN_ and MILLA_NONLIN_, compared with those areas that appear in at least one model: MIDA–Duke (A), MIKE_LIN_–Duke (B), MIKE_NONLIN_–Duke (C), MIDA–Ella (D), MILLA_LIN_–Ella (E), and MILLA_NONLIN_—Ella (F)

Figure 4 shows the mean difference in |E‐field|^2^ for Duke–MIKE and Ella–MILLA for the cases of LIN and NONLIN registration. The calculation was made in the regions present in the two models being compared. Thus, the biggest differences were found in the locations corresponding to the unmatched regions.

### 3.3 Effects of subject head morphometry in CP mode

**FIGURE 5:**
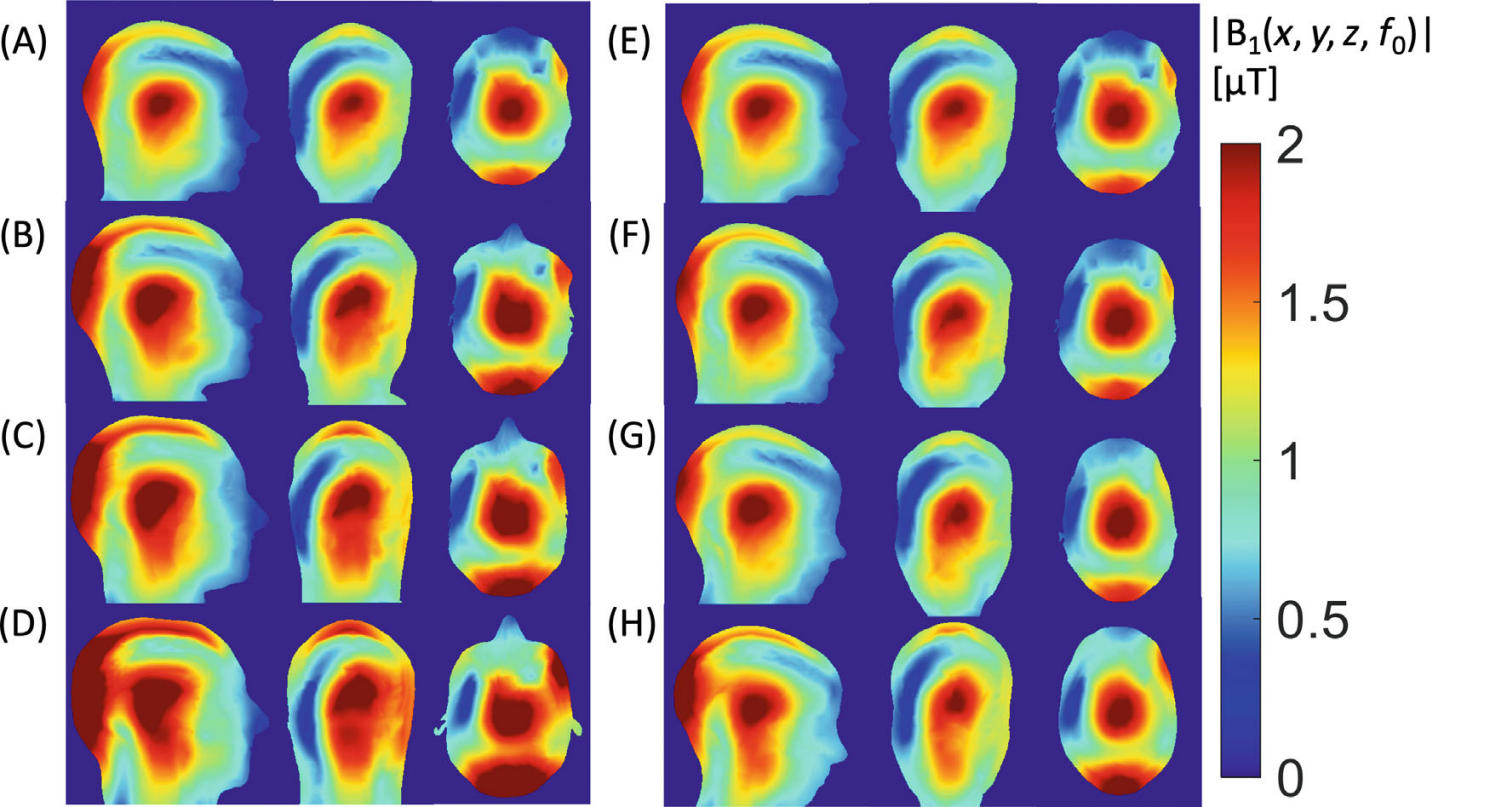
B_1_^+^ field in circularly polarized (CP) mode (normalized to 2 μT at the coil center) in coronal, sagittal, and axial views of MIDA model (A), MIKE_LIN_ model (B), MIKE_NONLIN_ model (C), Duke model (D), MIDA model (E), MILLA_LIN_ model (F), MILLA_NONLIN_ model (G), and Ella model (H)

Figure 5 shows the B_1_ transmit field calculated across the different models in CP mode. When estimating the B_1_^+^ field distribution in Duke (Figure 5D) and Ella (Figure 5H) from the reference model MIDA (Figure 5A,E), there was broad agreement when affine LIN registration was used to align the MIDA model (Figure 5B,F). This was further improved when NONLIN registration was used to better match details of the head morphometry between the reference and target voxel models (Figure 5C,G). RMS error in the B_1_^+^ field in the head between the MIDA and Duke models was reduced from 5.87 to 3.80 μT (i.e., 35.3% improvement) when affine LIN registration was applied, and further reduced to 2.58 μT (55.9% improvement) when NONLIN registration was used. In the case of MILLA, the mean difference in B_1_^+^ field between MIDA and Ella was 3.53 μT, which was reduced to 2.92 μT (17.4% improvement), and 2.26 μT (36.1% improvement) in the case of affine LIN and NONLIN registration, respectively.

The SAR_head_ and the maximum 10gSAR in CP mode are given in Table 2. The difference in SAR_head_ estimation between the MIDA and Duke models' head positioned at the coil center was 41.3%, which was reduced to 25.8% when the affine LIN registration was applied to MIDA, and further reduced to 19.2% when NONLIN registration was applied to align MIDA into Duke space. The difference in SAR_head_ estimation between MIDA and Ella was 25.0% when the models' head was positioned at the coil center, and 20.7% and 18.1% in the case of affine LIN and NONLIN registration, respectively.

**TABLE 2:** Head‐averaged SAR and 10gSAR in CP mode (fields were normalized to 2 μT at the coil center). Averaged SAR in the head region for each model is shown in the first row; worst‐case 10gSAR for each model is shown in the second row; normalization factor (V) is shown in the third row; and total absorbed power in the head (W) is shown in the last row

	MIDA	Duke	MIKE_LIN_	MIKE_NONLIN_	Ella	MILLA_LIN_	MILLA_NONLIN_
SAR_head_ (W/kg)	1.41	2.29	1.70	1.89	1.81	1.44	1.49
10gSAR_max_ (W/kg)	4.87	8.34	6.43	7.37	5.44	4.62	4.82
Normalization factor (V)	1.41	1.95	1.61	1.70	1.67	1.44	1.45
Total absorbed power_head_ (W)	6.28	11.69	8.34	9.35	7.69	6.18	6.23

**FIGURE 6:**
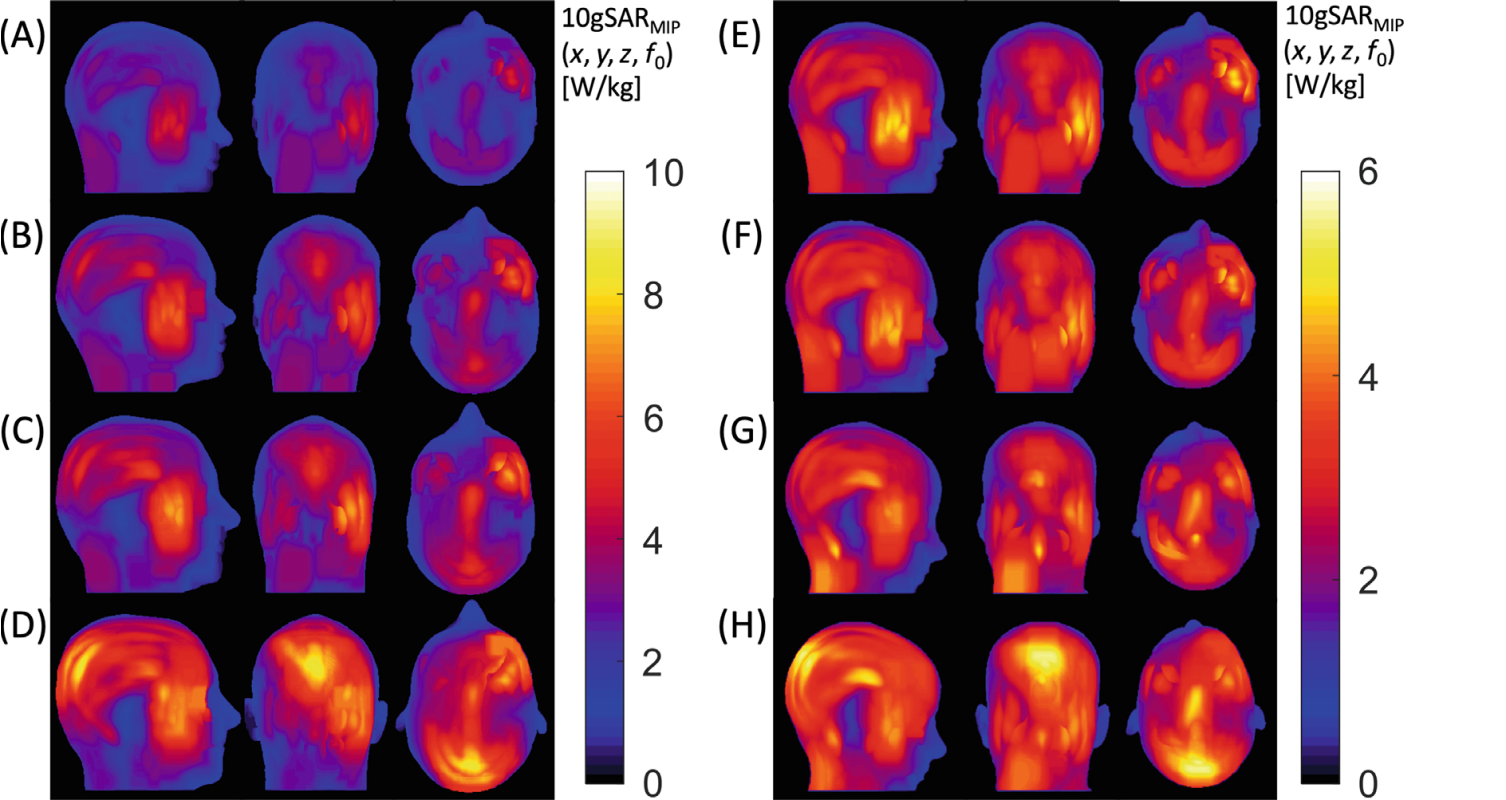
Maximum intensity projection of 10‐g mass averaged SAR (10gSAR) within the head region in CP mode (fields were normalized to 2 μT at the coil center) in sagittal, coronal, and axial view of MIDA model (A), MIKE_LIN_ model (B), MIKE_NONLIN_ model (C), Duke model (D), MIDA model (E), MILLA_LIN_ model (F), MILLA_NONLIN_ model (G), and Ella model (H)

The results of the 10gSAR in CP mode with maximum intensity projection are shown in Figure 6. In the case of Duke versus MIDA with the head positioned at the coil center, the difference in maximum 10gSAR was 41.6%, which was reduced to 22.9% when affine LIN registration was applied to register the MIDA model into Duke space, and further reduced to 11.6% when NONLIN registration was applied to warp the MIDA model into the Duke head morphometry. Interestingly, the difference in maximum 10gSAR between MIDA and Ella was only 10.5% initially, which increased to 15.1% when the affine LIN registration was applied to register MIDA into Ella space, and 11.4% (a marginal increase from the initial difference) when NONLIN registration was applied to warp the MIDA model into Ella space.

### 3.4 Estimation of maximum pTx 10gSAR in 5000 random RF shim sets

**FIGURE 7:**
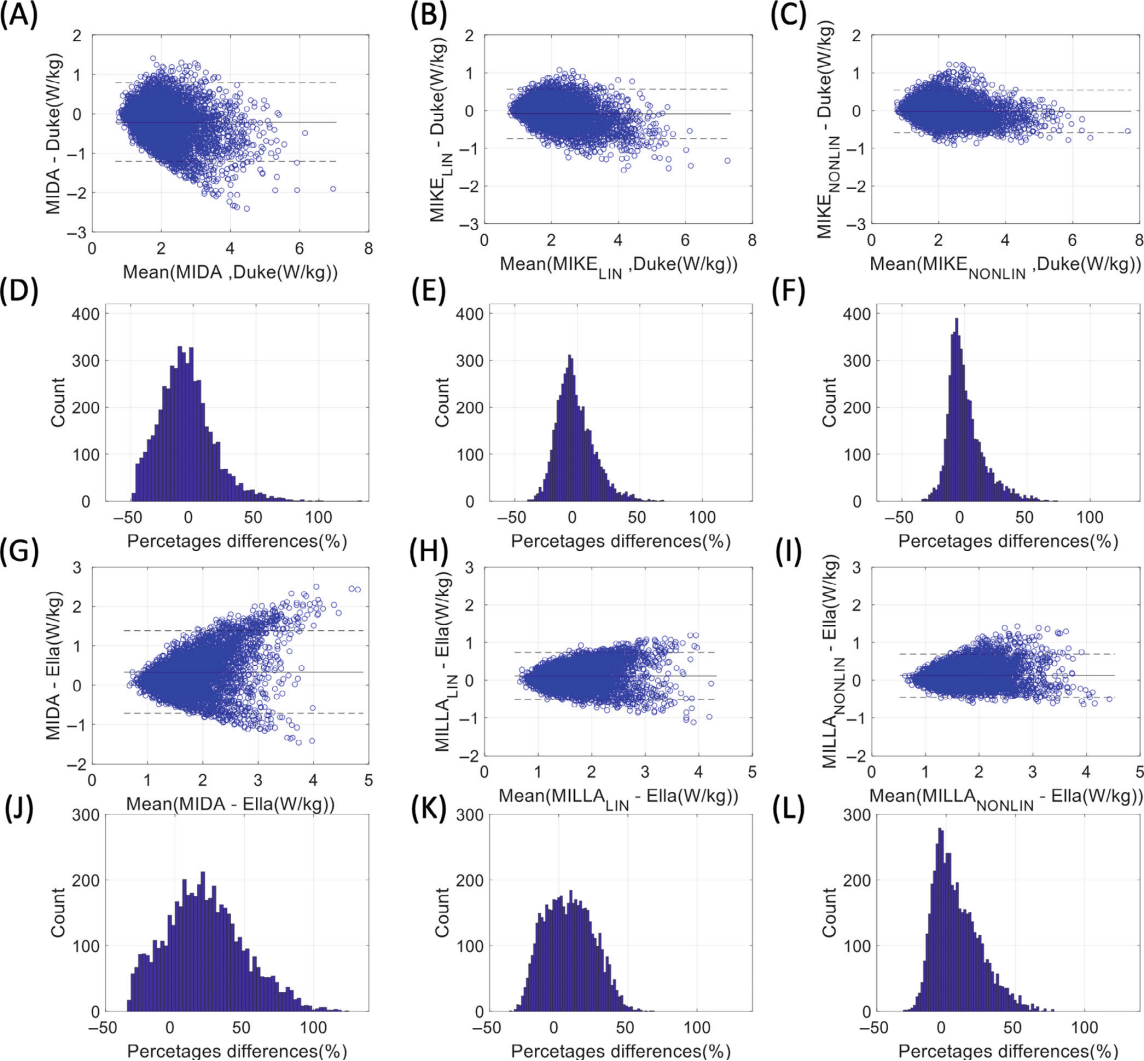
Bland–Altman plots comparing the highest local 10gSAR predicted by Duke to that predicted by MIDA (with and without warping) for 5000 random B_1_^+^ shim sets. The mean difference is shown as a solid line parallel to the *y*‐axis, and the standard deviation in each direction is shown as dashed lines. (A) MIDA—Duke; (B) MIKE_LIN_—Duke; (C) MIKE_NONLIN_—Duke; (G) MIDA—Ella; (H) MILLA_LIN_—Ella; (I) MILLA_NONLIN_—Ella. Also shown are histograms of maximum 10gSAR difference between (D) MIDA—Duke; (E) MIKE_LIN_—Duke; (F) MIKE_NONLIN_—Duke; (J) MIDA—Ella; (K) MILLA_LIN_—Ella; (L) MILLA_NONLIN_—Ella. SAR, specific absorption rate

The results of calculating the maximum 10gSAR value in the various different models when using 5000 randomly chosen RF shim sets are shown in Figure 7. The Bland–Altman plots show the mean and difference in maximum 10gSAR for each shim set between two particular models (e.g., Duke target model vs. the MIDA‐warped‐into‐Duke model, MIKE_NONLIN_). The initial difference in maximum 10gSAR prediction in 5000 RF shim sets are shown in Figure 7A for MIDA versus Duke, and in Figure 7G for MIDA versus Ella. The mean absolute differences in maximum 10gSAR was 0.40 and 0.48 W/kg, for Duke and Ella, respectively. When affine LIN registration was applied to align MIDA‐into‐Duke or MIDA‐into‐Ella, the mean absolute difference in maximum 10gSAR was reduced in both cases to 0.26 W/kg. In the case of NONLIN registration of the MIDA model, the mean absolute difference in maximum 10gSAR was further reduced to 0.22 W/kg in the case of MIDA‐warped‐into‐Duke and 0.23 W/kg in the case of MIDA‐warped‐into‐Ella. Histograms of percent difference in maximum 10gSAR are shown in Figure 7D,F in the case of MIDA‐warped‐into‐Duke, and in Figure 7J,L for MIDA‐warped‐into‐Ella for no registration, LIN registration, and NONLIN registration, respectively. The mean absolute percent difference in maximum 10gSAR estimation over 5000 random RF shim sets was 17.5% for MIDA versus Duke and 28.3% for MIDA versus Ella. This mean percent difference in maximum 10gSAR was reduced to 11.8%, and 15.3% when the affine LIN registration was applied to MIDA‐into‐Duke (MIKE_LIN_) and MIDA‐into‐Ella (MILLA_LIN_), respectively. The mean percent difference in maximum 10gSAR was further reduced when NONLIN registration was applied to MIDA‐into‐Duke (MIKE_NONLIN_) and MIDA‐into‐Ella (MILLA_NONLIN_), giving values of 10.7%, and 13.9%, respectively. These results were consistent when VOP compression was used (Figure S5).

### 3.5 Uncertainty in the maximum 10gSAR estimation in the head from segmentation

**FIGURE 8:**
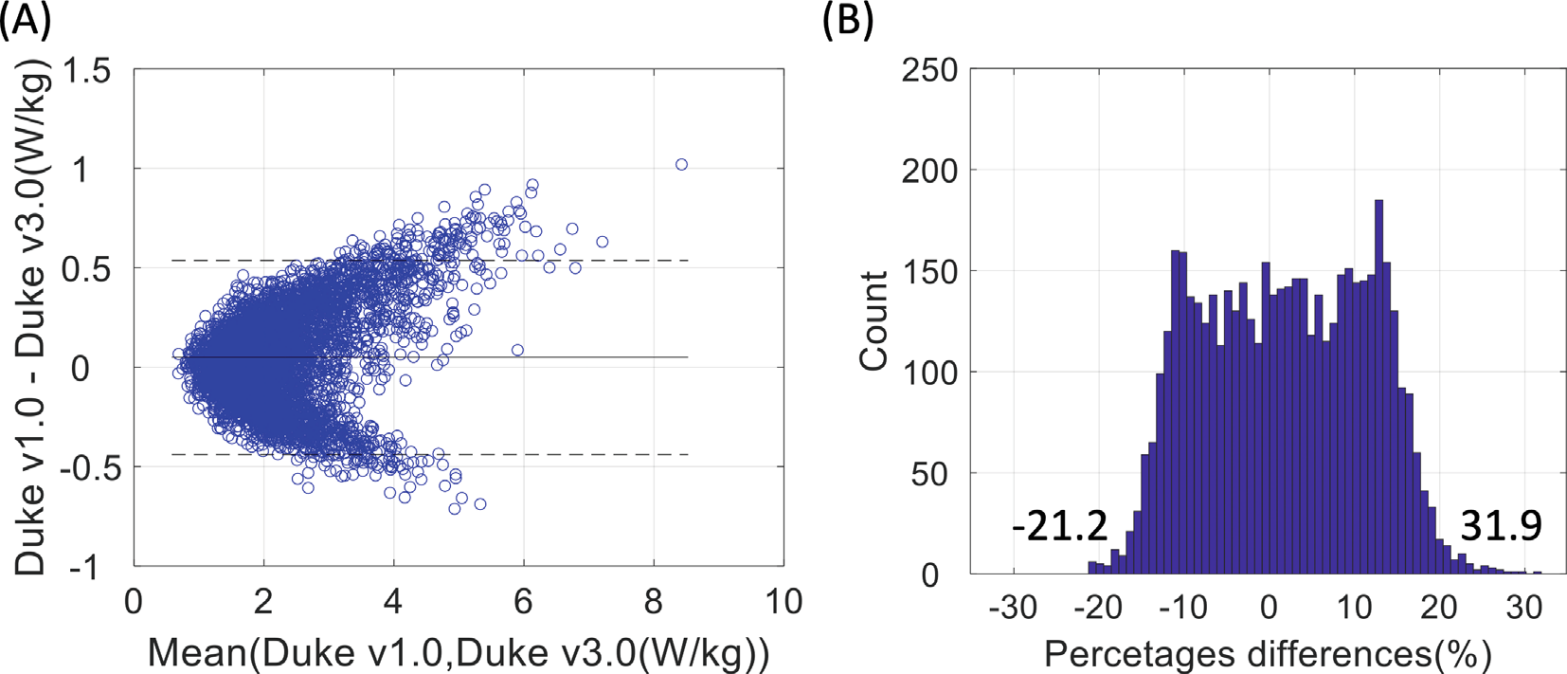
Uncertainty analysis. A, Bland–Altman plot comparing the maximum local SAR predicted by Duke version 1.0 to that predicted by Duke version 3.0 for each B_1_^+^ shim set. B, Histogram of maximum 10gSAR differences between Duke version 1.0 versus Duke version 3.0

The results of maximum 10gSAR estimation in 5000 random RF shim sets applied to Duke version 1.0 versus version 3.0 are shown in Figure 8. The mean absolute difference in maximum 10gSAR was 0.20 W/kg, and the mean absolute percent difference in maximum 10gSAR was 8.4%. The full range in maximum 10gSAR between Duke version 1.0 and version 3.0 was found to be 31.9% (16.4% difference when considering the 95th percentile difference), as shown in Figure 8. These data show the potential degree of uncertainty caused by segmentation differences, even in the same native subject.

## 4 DISCUSSION

In this study, we have evaluated via numerical simulation the effects of head morphometry and use of a registered “reference” EM model in RF safety estimation for 7T pTx MRI of the head. The use of pTx for 7T MRI is a promising technique to mitigate B_1_^+^ inhomogeneity, leading to enhanced uniformity in the resulting image. However, the risks related to RF heating need to be assessed carefully to realize the benefits of 7T pTx MRI for diagnosis and neuroscience research. 7T MRI is currently cleared by the US Food and Drug Administration for clinical use in the head and limbs of patients who weigh more than 66 lbs. (30 kg).^29^ Thus, there remains a need to understand the risks related to RF heating in a clinical scan. The following sections describe our observations on the effect of head morphometry in 7T head MRI RF safety estimation.

### 4.1 LIN versus NONLIN registration in subject‐specific RF safety estimation

Affine LIN registration of a reference model's brain (e.g., MIDA) into a target model's brain (e.g., Duke or Ella) showed an improvement in the estimation of the subject‐specific SAR over a simple head‐centered positioning of the reference model. The main effect gained by the affine LIN registration was improved accuracy of the relative position and size of the target model inside the RF transmit coil. Although the resulting estimation of subject‐specific maximum 10gSAR for LIN registration was not as accurate as NONLIN registration, there are benefits of LIN registration over NONLIN registration. First, it is relatively easy to implement on a reference voxel model if one can obtain brain‐only information using a brain extraction analysis tool. Second, the calculation and processing time for affine LIN registration was achieved within 4 min (5 s for brain extraction, and 3 m 45 s for the LIN registration running on a 3.5‐GHz dual‐core Intel i7 CPU with 16 GB LPDDR3 memory). An affine LIN registration approach could also potentially be used in conjunction with T_1_‐weighted brain images extracted from large‐population MR data sets (e.g., UK Biobank brain imaging^30^ or Human Connectome project^31^) to assess population variability. Precalculated head SAR maps on such data sets could then be compressed to create SAR monitoring matrices (e.g., VOP^27^, ^28^), to account for patient head position or to determine RF safety margins across a population for specific coils and magnetic field strengths (Figure S6).

NONLIN registration showed an improved estimation of target model head SAR estimation compared with affine LIN registration. However, the NONLIN warping field calculation time took a total of 27 min (approximately 4 min for the LIN registration preprocessing step to find the initial position and an additional 23 min for NONLIN registration using the same computer as previously). In this regard, the NONLIN registration method is currently unsuitable for implementation in rapid SAR calculation. Another challenge of NONLIN registration using the MIDA model as the reference is the lack of a synthesized T_1_‐weighted image of the entire head and shoulders (e.g., down to the level of the T5 vertebrae). Thus, the MIDA model may not be suitable for applications that require imaging below the level of the base of the brain, or that use RF transmit coils with a larger coverage. A dedicated “reference” scan that included the entire head and shoulders might also be of interest to further study the effects of head morphometry in SAR estimation, as well as for other computational modeling studies such as EM stimulation for neuroprotective treatments^32^ and hyperthermia treatment planning in patients with cancer.^33^, ^34^

### 4.2 Effects of head morphometry in head SAR estimation of 7T MRI

The proposed method showed the potential to estimate more consistent results with the target model simulation to account for differences in patient head morphometry across populations. Using LIN and NONLIN registration to estimate head SAR in CP mode, NONLIN warping of the head morphometry to a “reference” model has already shown a marginal effect as previously reported by Jin et al.^20^ In our studies, the warping method when applied to Ella in CP mode showed that the benefit is marginal or low if the initial error is within the uncertainty range (i.e., ± 20% for the 95th percentile with a maximum of 34% estimated in this study for the non–head morphometry–related aspects; see Figure 8). This may in part be due to the inherent similarity of these two female head models. The benefits of matching head morphometry were more prominent when considering RF safety estimation in the case of pTx MRI. According to the Milshteyn et al. study,^16^ the intervolunteer variation of maximum 10gSAR for 3T head MRI is 20.3% when a birdcage body transmit coil is used. Le Garrec et al.^35^ suggested that a safety margin of 1.5 (50%) accounts for intersubject variability in MRI RF safety assessment, ensuring a < 1% chance of exceeding the corresponding SAR limit. Translating that approach to this study, the averaged absolute maximum 10gSAR error range between Duke and MIDA at the 99th percentile was found to be 16.7% when models' heads are positioned at the coil center, which reduced to 11.2% when affine LIN registration was used (MIKE_LIN_) and 10.1% when NONLIN registration was used (MIKE_NONLIN_). The corresponding figures for Ella versus MIDA were 27.5% at the initial position, 14.8% for affine LIN registration (MILLA_LIN_), and 13.3% for NONLIN registration (MILLA_NONLIN_).

### 4.3 Uncertainties in tissue composition in the voxel models: effects of tissue composition

The details and variety of the tissue compartments in the voxel models may vary depending on the segmentation tool used for model development, or the intended application, or the imaging modality used. The MIDA, Duke, and Ella models are adult models that are used widely in computational modeling. However, it is important to note that there are inherent differences in tissue labeling, segmentation, and number of compartments among these various models. The effect of tissue composition has been studied previously. According to Wolf et al.,^36^ when tissue became more uniform there was more uniform SAR deposition. Tissue simplification removes the transitions at dielectric boundaries between adjacent tissues, which limits increased local SAR and keeps hotspots stable.^36^ To help assess the uncertainties in tissue composition that were not related to head morphometry, the maximum 10gSAR values across 5000 random B_1_ shim sets between Duke version 1.0 and Duke version 3.0 were compared (Figure. 8). Both versions of the Duke model were developed from medical images from the same subject. Thus, the detailed morphometry is expected to be identical. The Duke version 3.0 model is a newer version that was updated with improved quality control procedures and numerous anatomical refinements 4 years after the initial Duke version 1.0 production (Figure S4 and Table S1). Both lower and higher SAR is evident in Duke version 1.0 compared with version 3.0, which could potentially be due to the introduction of new dielectric boundaries. For example, some of the low conductive tissue such as cortical bone in Duke version 1.0 was further segmented to have a higher conductive tissue boundary of cancellous bone inside. Transition to a higher conductivity tissue could induce higher SAR.^36^ Conversely, dura mater was added to the model, which has a lower conductivity compared with CSF, and therefore is expected to lower the SAR in the segmented region. The maximum 10gSAR across these two different versions of the Duke model resulted in a 26.4% difference in 10gSAR estimation, which is independent of head morphometry. This comparison highlights that a significant degree of uncertainty, on the order of 25%, remains in 7T pTx MRI head SAR estimation that can be ascribed to the accurate characterization of the target model's tissue composition.

### 4.4 Alternative methods

An alternative method to achieve a subject‐specific model is to attempt a direct segmentation of the tissue. Automated segmentation tools, such as headreco in SimNIBS,^17^ allow one to segment a list of head tissues using multicontrast MRI data (e.g., T_1_‐weighted and T_2_‐weighted images), resulting in accurate skin, CSF, skull, brain gray matter and white matter and cerebellum, which can be integrated into FSL or *SPM12* as part of an automated processing pipeline. Thus, a personalized voxel model for computational simulation can be generated with minimum anatomical knowledge. However, the processing time for the headreco in SimNIBS (an automated skull segmentation tool) is very long (estimated to be approximately 3 h for a 0.5 × 0.5 × 0.5 mm resolution data set using the same computer hardware as previously). Furthermore, the automated skull segmentation tool was initially developed to account for the upper skull and brain components to estimate the time‐varying magnetic field interaction in applications such as transcranial magnetic stimulation. Thus, manual refinement to calculate SAR in the head is still needed for non‐brain structures, such as the mandible and neck, and to attach a model of the shoulders and trunk to avoid EM boundary artifacts.

Alternatively, recent advances in machine learning in medical image segmentation techniques have greatly improved the segmentation processing time. For example, a commercial segmentation tool has demonstrated that deep brain structures could be segmented within 5 min using an initial T_1_‐weighted MRI.^37^ As such, a deep learning–based segmentation tool could be a potential technique to achieve personalized safety modeling.

For real‐time SAR calculation, segmentation with a reduced number of tissue compartments could be used.^16^ Previously, de Buck et al. reported that a strategic clustering of tissue compartments in the head can lead to an error in maximum 10gSAR estimation in pTx head MRI of less than 12% compared with the full number of tissue compartments of voxel models from different age groups in the virtual population.^38^ However, the range of error reported in the de Buck et al. study did not fully account for deviation from segmentation at boundaries between tissue compartments.

### 4.5 Limitations

There are a number of potential limitations of this study. First, the simulation is only tuned once for the head‐centered MIDA model with an assumption that transmit coils do not have an automated tuning capacity.^39^ The reflection coefficient (i.e., S‐matrix) values are caused by interactions between the transmit coil elements and the loaded sample at each port in the simulation; thus, their values vary among models (Figure S7). According to Restivo et al.,^40^ differences in the predicted maximum 10gSAR can vary by up to 41% in phantom studies due to variation in S‐matrices (magnitude and phase). Second, our study was conducted using only three representative male and female voxel models, which may not account for the head morphometry differences across the wider population, including other ethnic groups. Third, our proposed process requires a manual step to attach a body from open‐source whole‐body models. An automated body attachment process might be useful for the proposed application, but it has not been further investigated in this work. Fourth, none of the models or simulations in this work were validated in vivo. Finally, this study focused on a single, specific eight‐channel loop array coil (Affinity Imaging). The results may vary for a different number of transmit coils, arrangement, or type of transmit coil, such as dipole arrays.^41^ The current IEC standard suggests RF heating–related limits in both SAR and temperature. However, this study only analyzed the potential local SAR for 7T pTx head imaging.

## 5 CONCLUSIONS

We have presented affine LIN and NONLIN registration methods to assess the effects of head morphometry in subject‐specific RF safety estimation of 7T pTx head MRI. The study demonstrates that SAR prediction, which is more consistent with a target model simulation, is possible by matching brain size, position, and morphometry to an existing EM reference model via a target model's T_1_‐weighted images. However, head morphometry alone is unable to account for differences among models, with remaining differences likely caused by tissue segmentation. This suggests that the accurate estimation of tissue composition plays an important role as well as the matching of the head morphometry when high‐resolution models are used to estimate patient‐specific SAR in 7T pTx head MRI. Thus, the uncertainty of head morphometry and tissue composition—whether the voxel models are simplified or an alternative version of the same model is used in SAR assessment—needs to be considered to achieve personalized SAR estimation.

## AUTHOR CONTRIBUTIONS

*Software*: Hongbae Jeong, Jesper Andersson, and Aaron Hess. *Investigation, manuscript draft, and visualization*: Hongbae Jeong. *Reviewing and editing*: Jesper Andersson, Aaron Hess, and Peter Jezzard. *Methodology, writing, study concept, and supervision*: Aaron Hess and Peter Jezzard.

## CONFLICT OF INTEREST

Peter Jezzard is the editor‐in‐chief of *Magnetic Resonance in Medicine*. In line with COPE guidelines, he recused himself from all involvement in the review process of this paper, which was handled by an associate editor. He and the other authors have no access to the identity of the reviewers.

## Supporting information


**Figure S1.** A, Command lines for linear and nonlinear registration of voxel‐based model. B, Code lines to register a CAD‐based model to NIfTI space
**Figure S2.** Body attachment process of the multimodal image‐based detailed anatomical (MIDA) head‐only model. A, The MIDA head model is aligned onto an open‐source male body model. B, Extruded area of the neck in the body model was dragged inward using drag function in the sculpt tool. C, Connection between head and body model is smoothed (bubble smooth function in sculpt tool) and pinched (pinch function in sculpt tool) to have a natural connection. D, Skin of the head and body model is combined, and any extruded element of subcutaneous adipose tissue (SAT) and muscle is reduced using the same process as explained previously
**Figure S3.** Code lines to combine multiple simulations for use with the calculation of 5000 random B_1_ shim sets
**Figure S4.** The fidelity of voxel electromagnetic (EM) properties. A,B, Conductivity differences of Duke v3.0 (Duke v1.0 in coronal view) (A) and axial view (B). C,D, Tissue density differences of Duke v3.0 (Duke v1.0 in coronal view) (C) and axial view (D)
**Figure S5.** Compressed results for the Bland–Altman plots comparing the highest local 10‐g mass averaged specific absorption rate (10gSAR) predicted by Duke to that predicted by MIDA (with and without warping) at maximum 5% overestimation for 5000 random B_1_^+^ shim sets. A, MIDA—Duke. B, MIKE_LIN_—Duke. C, MIKE_NONLIN_—Duke. G, MIDA—Ella. H, MILLA_LIN_—Ella. I, MILLA_NONLIN_—Ella. Also shown are histograms of maximum 10gSAR difference among MIDA—Duke (D), MIKE_LIN_—Duke (E), IKE_NONLIN_—Duke (F), MIDA—Ella (J), MILLA_LIN_—Ella (K), and MILLA_NONLIN_—Ella (L). See Figure 7 for the uncompressed results for the Bland–Altman plots
**Figure S6.** Effects of linear registration compared with nonlinear registration. A, Bland–Altman plot comparing the hottest local SAR predicted by affine linear registration of MIDA into Duke (MIKE_LIN_) versus that predicted by nonlinear registration (MIKE_NONLIN_) for 5000 B_1_^+^ shim sets. B, Histogram of maximum 10gSAR differences between linear registration of MIDA to Duke versus nonlinear registration. C,D, The corresponding plots for affine linear registration of MIDA into Ella (MILLA_LIN_) versus that predicted by nonlinear registration (MILLA_NONLIN_)
**Figure S7.** Simulated S‐parameters in dB. A, MIDA model. B, Duke model. C, MIKE_LIN_ model. D, MIKE_NONLIN_ model. E, Ella model. F, MILLA_LIN_ model. G, MILLA_NONLIN_ model
**Table S1.** Tissue properties at 297.2 MHz: tissue label only existing in Duke v1.0 (A); tissue labels existing in both Duke v1.0 and v3.0 that were further separated in Duke v3.0 (B); and tissue labels only existing in Duke v3.0 (C)
