# Supplementary material for: Effect of subject‐specific head morphometry on specific absorption rate estimates in parallel‐transmit MRI at 7 T
Source: Magn Reson Med. 2023 Jan 19;89(6):2376–90. doi: 10.1002/mrm.29589 (PMC10952207; doi:10.1002/mrm.29589)
Supplement: Supplementary file 1 — Figure S1. A, Command lines for linear and nonlinear registration of voxel‐based model. B, Code lines to register a CAD‐based model to NIfTI space Figure S2. Body attachment process of the multimodal image‐based detailed anatomical (MIDA) head‐only model. A, The MIDA head model is aligned onto an open‐source male body model. B, Extruded area of the neck in the body model was dragged inward using drag function in the sculpt tool. C, Connection between head and body model is smoothed (bubble smooth function in sculpt tool) and pinched (pinch function in sculpt tool) to have a natural connection. D, Skin of the head and body model is combined, and any extruded element of subcutaneous adipose tissue (SAT) and muscle is reduced using the same process as explained previously Figure S3. Code lines to combine multiple simulations for use with the calculation of 5000 random B1 shim sets Figure S4. The fidelity of voxel electromagnetic (EM) properties. A,B, Conductivity differences of Duke v3.0 (Duke v1.0 in coronal view) (A) and axial view (B). C,D, Tissue density differences of Duke v3.0 (Duke v1.0 in coronal view) (C) and axial view (D) Figure S5. Compressed results for the Bland–Altman plots comparing the highest local 10‐g mass averaged specific absorption rate (10gSAR) predicted by Duke to that predicted by MIDA (with and without warping) at maximum 5% overestimation for 5000 random B1 + shim sets. A, MIDA—Duke. B, MIKELIN—Duke. C, MIKENONLIN—Duke. G, MIDA—Ella. H, MILLALIN—Ella. I, MILLANONLIN—Ella. Also shown are histograms of maximum 10gSAR difference among MIDA—Duke (D), MIKELIN—Duke (E), IKENONLIN—Duke (F), MIDA—Ella (J), MILLALIN—Ella (K), and MILLANONLIN—Ella (L). See Figure 7 for the uncompressed results for the Bland–Altman plots Figure S6. Effects of linear registration compared with nonlinear registration. A, Bland–Altman plot comparing the hottest local SAR predicted by affine linear registration of MIDA into Duke (MIKELIN) versus that predicted by nonlinear [file MRM-89-2376-s001.docx]

# Supporting Information

# The effect of subject-specific head morphometry on specific absorption rate estimates in parallel transmit MRI at 7T

Hongbae Jeong^1,2^, Jesper Andersson^1^, Aaron Hess^1,3,4^, and Peter Jezzard^1^

^1^ Wellcome Centre for Integrative Neuroimaging, FMRIB Division, Nuffield Department of Clinical Neurosciences, University of Oxford, Oxford, United Kingdom.

^2^ Athinoula A. Martinos Center for Biomedical Imaging, Department of Radiology, Massachusetts General Hospital, Boston, MA, USA

^3^ Centre for Clinical Magnetic Resonance Research, Department of Cardiovascular Medicine, University of Oxford, Oxford, United Kingdom

^4^ British Heart Foundation Centre for Research Excellence, Oxford, United Kingdom

# S1. Code used for linear and non-linear registration

The following figures show the command lines used for linear and non-linear registration. To make it concrete, the example pertains to warping MIDA model to Duke model, but would apply equally well to MIDA model to Ella model.

| **# Starting input are MIDA_T1 and DUKE_T1. These are the MIDA and DUKE models**  **# respectively after having been converted into synthetic T1 images by assigning**  **# intensity values to the various structures in the models.**  **# There are also brain extracted (images where only brain tissue have non-zero**  **# intensity) versions named MIDA_T1_brain and DUKE_T1_brain respectively.**  **# Down-sampling; eye.mat is the 4 × 4 identity matrix. “refspace” is an empty**  **# image with the same FOV as DUKE_T1, but with 2mm cubed voxel size.**  applywarp --in=DUKE_T1 --ref=refspace --super --superlevel=4 --interp=spline  --premat=eye.mat --out=DUKE_T1_2mm  **# Downsample the _brain image in the same way**  **# Brain was created by setting the intensity of all non-brain tissue to zero**  applywarp --in=DUKE_T1_brain --ref=refspace --super --superlevel=4 --interp=spline  --premat=eye.mat --out=DUKE_T1_brain_2mm  **# Linear registration (calculating an affine transform matrix)**  flirt -ref DUKE_T1_brain_2mm -in MIDA_T1_brain -omat MIDA2DUKE.mat  -out MIKE_Lin  **# Non-linear registration (calculating warp-field)**  fnirt --ref=DUKE_T1_2mm --in=MIDA_T1 --aff=MIDA2DUKE.mat  --config= T1_2_Fake_T1_2mm.cnf --cout=MIDA2DUKE_warps --iout=MIKE_Nonlin  --fout=MIDA2DUKE_field -v  **% config=T1_2_Fake_T1_2mm.cnf**  --ref=Fake_T1_2mm  --imprefm=0  --impinm=0  --imprefval=0  --impinval=0  --subsamp=4,4,2,2,1,1  --miter=5,5,5,5,5,10  --infwhm=8,6,5,4.5,3,2  --reffwhm=8,6,5,4,2,0  --lambda=300,150,100,50,40,30  --estint=1,1,1,1,1,0  --applyrefmask=0  --applyinmask=0  --warpres=10,10,10  --ssqlambda=1  --regmod=bending_energy  --intmod=global_non_linear_with_bias  --intorder=5  --biasres=50,50,50  --biaslambda=10000  --refderiv=0 |
| --- |
| Supporting Figure S1a: Command lines for linear and non-linear registration of voxel-based model. |

| 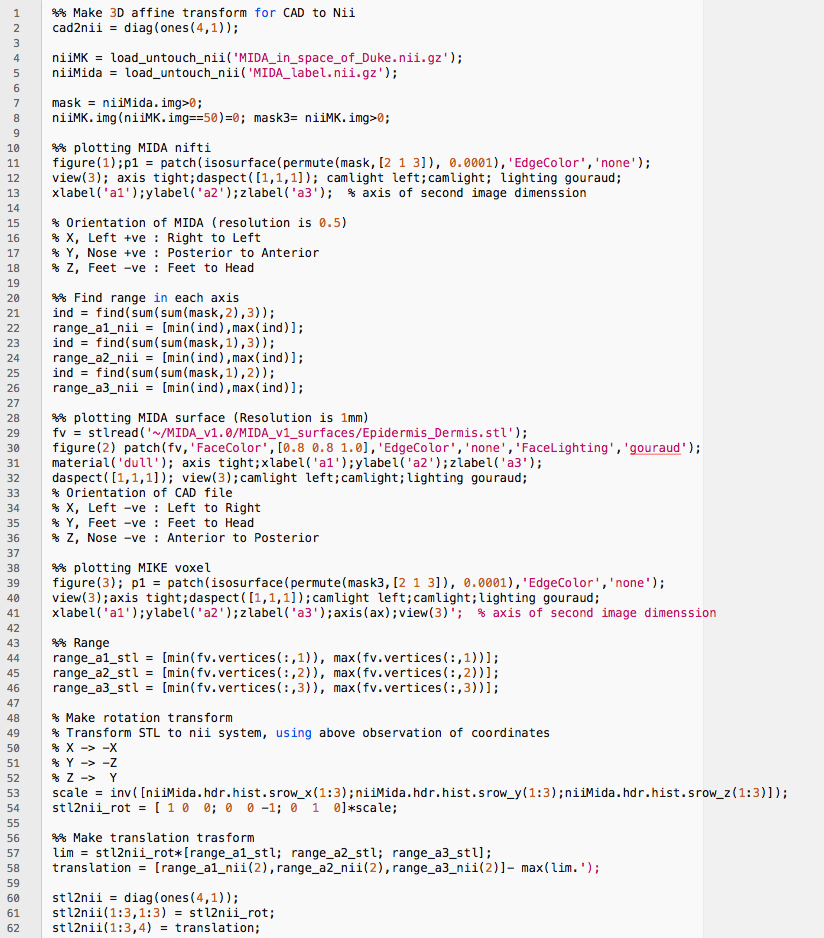 |
| --- |

| 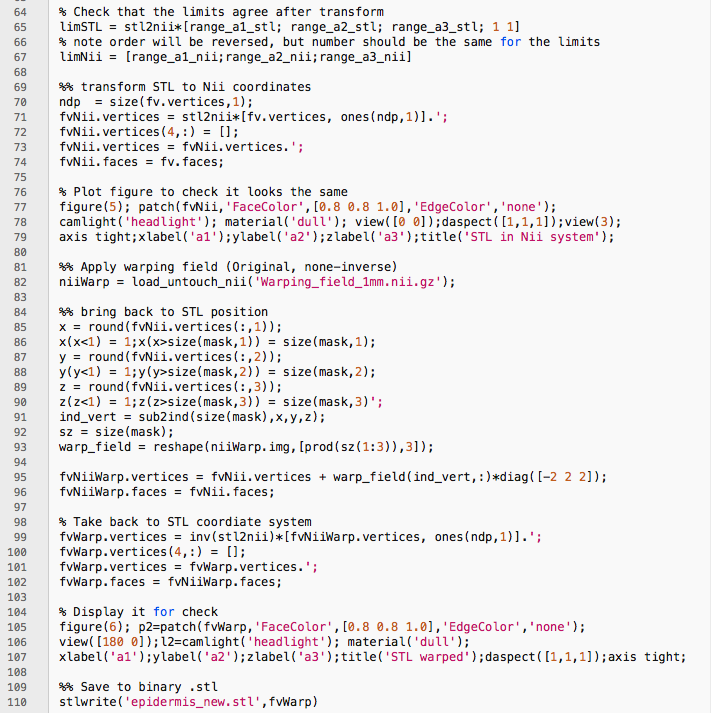 Supporting Figure S1b: Code lines to register a CAD-based model to nifti space |
| --- |

# S2. Process to attach a whole-body shoulder and trunk onto head-only model

This supplemental information contains the method used to attach the ETRI body model onto the MIDA head model to avoid electromagnetic boundary effects.

Supporting Figure S2 shows the process of body model attachment onto the MIDA head model using MeshMixer (Autodesk Inc., CA, USA).

| 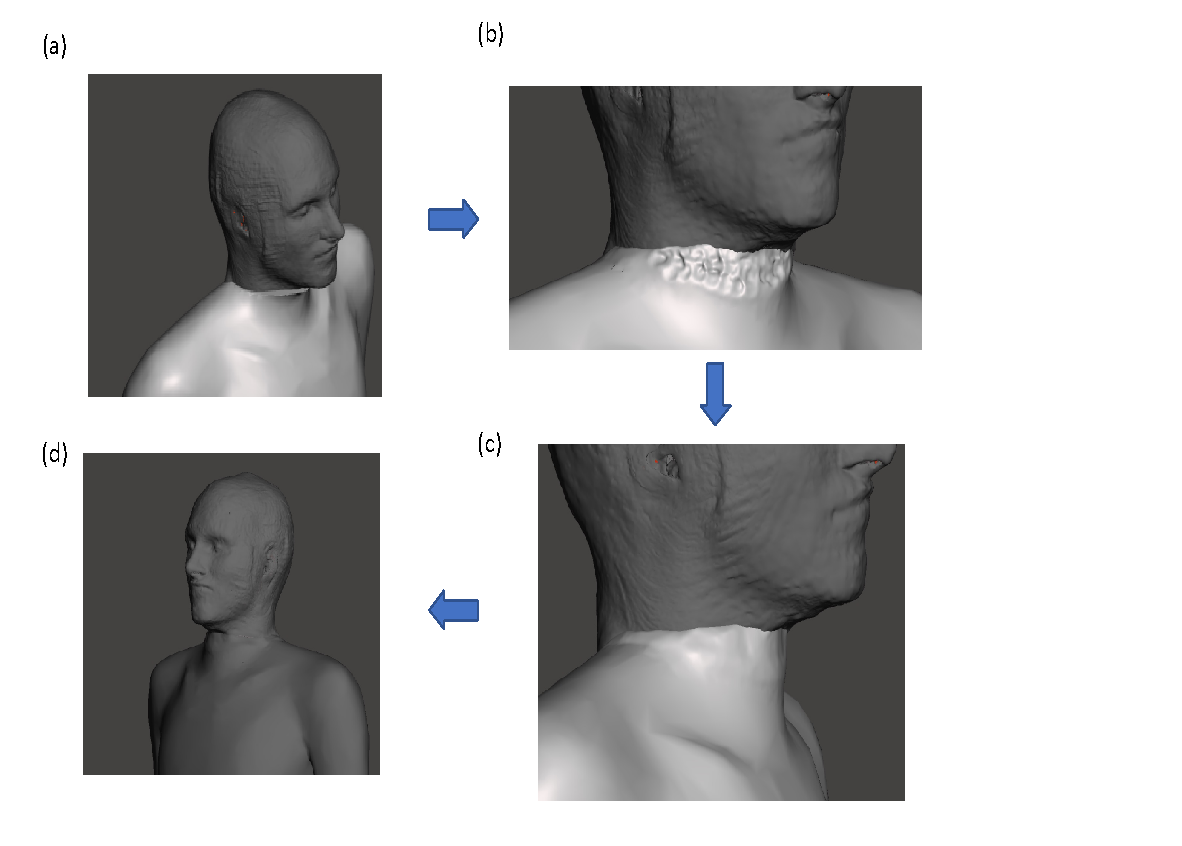 |
| --- |
| Supporting Figure S2: Body attachment process of MIDA head-only model. (a) The MIDA head model is aligned onto an open-source male body model; (b) Extruded area of the neck in the body model was dragged inward using drag function in the sculpt tool; (c) connection between head and body model is smoothed (bubble smooth function in sculpt tool) and pinched (pinch function in sculpt tool) to have a natural connection; (d) skin of the head and body model is combined and any extruded element of SAT and muscle is reduced using the same process as above. |

# S3. Code used for pTx field combiner

Below is a screenshot of the Python code used for the field combination step to generate pTx results. Code can be run in the Python scripter in Sim4Life.


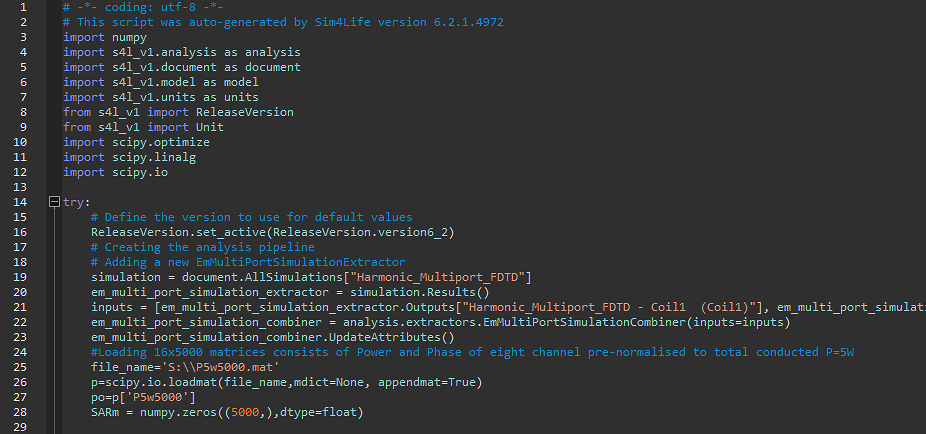


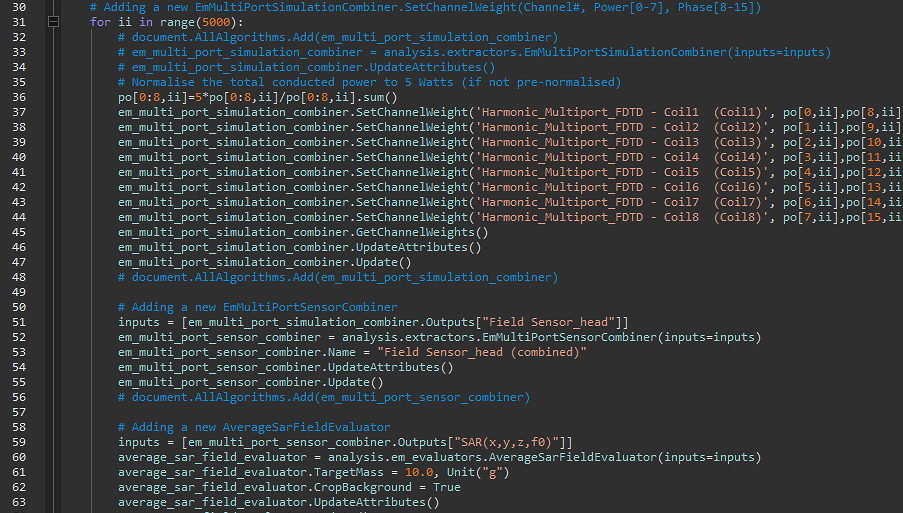


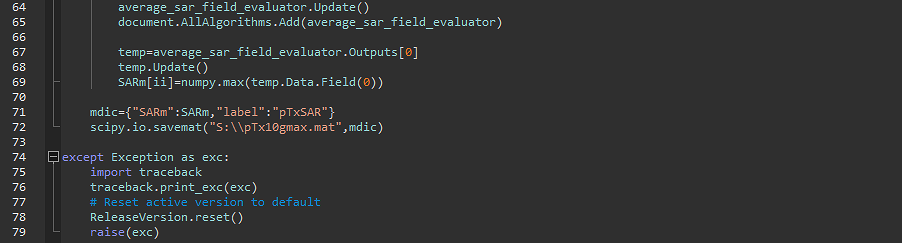


Supporting Figure S3: Code lines to combine multiple simulations for use with the calculation of 5,000 random B_1_ shim sets. (Total conducted power was pre-normalised to 5W in each set)

# S4. Tissue properties comparison between Duke v1.0 and Duke v3.0

Supporting Figure S4 shows the difference in conductivity and tissue density between Duke v1.0 (initially developed in 2010^1^) and Duke v3.0 (upgraded from Duke v1.0 in 2014^2^). The two models were developed based on medical images of the same subject, thus sharing the subject’s head morphometry. The v3.0 update includes the addition of new important tissue compartments, including the dura mater, cancellous bone, salivary gland, and the replacement of connective tissue with more appropriate tissue labels^2^. For example, the connective tissues in Duke v1.0, that was the label for unidentified tissue when Duke was initially developed in 2010, was replaced with the corresponding tissue label in Duke v3.0 with implementation of quality control procedures and numerous anatomical refinements^2^. Bone and bone marrow in Duke v1.0 were further segmented into cortical bone, bone marrow and cancellous bone (i.e., sponge bone) in Duke v3.0, and the cerebellum in Duke v1.0 was further divided into cerebellum and cerebellum white matter. Furthermore, the dura mater and the salivary gland were also added as new tissue labels in Duke v3.0. For further details of the update, please see the publication by Gosselin *et al.*^2^, and IT’IS Foundation website^3^. Supporting Table S-4 shows the tissue properties^4^ of the example tissue labels that are particularly available in Duke v1.0 and v3.0 models.

| 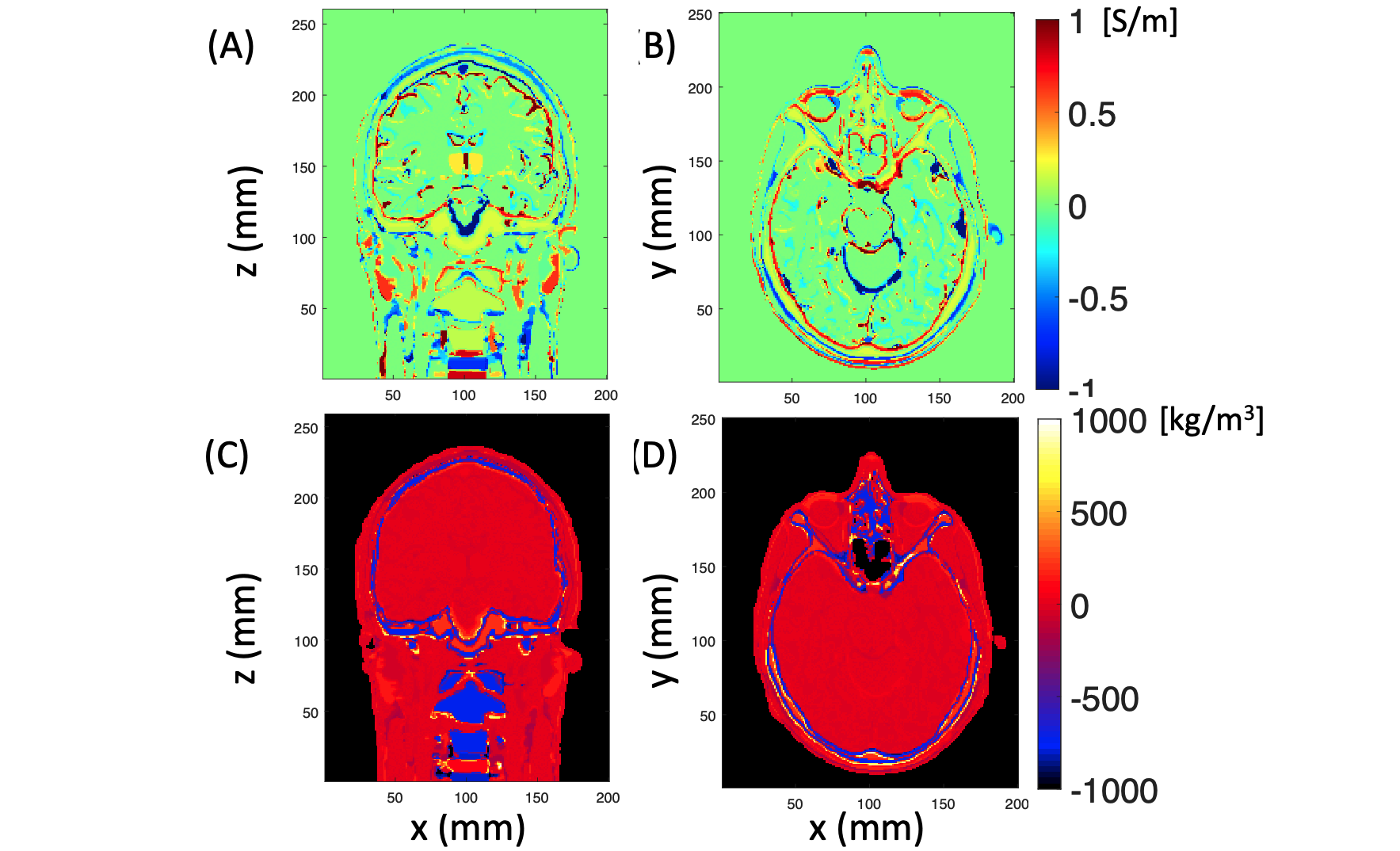 |
| --- |
| Supporting Figure S4: The fidelity of voxel EM properties: (A) Conductivity differences of Duke v3.0 – Duke v1.0 in coronal view; and (B) axial view; and (C) tissue density differences of Duke v3.0 – Duke v1.0 in coronal view; and (D) axial view. |
| \| Model version \| Tissue \| Density [kg/m^3^] \| Conductivity [S/m] \| Relative permittivity [a.u.] \| \| --- \| --- \| --- \| --- \| --- \| \| Frequency: 297.2 MHz \| \| \| 1. Duke v1.0 \| Connective tissue \| 1026.50 \| 0.54 \| 48.01 \| \| 1. Duke v1.0   &  Duke v3.0 \| Bone (Cortical) \| 1908.00 \| 0.08 \| 13.45 \| \| Bone marrow  (yellow) \| 980.00 \| 0.03 \| 5.76 \| \| Cerebellum \| 1045.00 \| 0.97 \| 59.86 \| \| 1. Duke v3.0 \| Dura \| 1174.00 \| 0.80 \| 48.01 \| \| Salivary gland \| 1048.00 \| 0.72 \| 78.00 \| \| Bone (Cancellous) \| 1178.33 \| 0.22 \| 23.19 \| \| Cerebellum WM \| 1041.00 \| 0.41 \| 43.84 \| \| Supporting Table S1: The tissue properties at 297.2 MHz, (A) tissue label only existing in Duke v1.0; (B) tissue labels existing in both Duke v1.0 and v3.0 that were further separated in Duke v3.0; (C) tissue labels only existing in Duke v3.0. \| \| \| \| \| |

# S5. Effect of compressed SAR model in 5,000 random B_1_^+^ shim sets

|  |
| --- |
| Supporting Figure S5: Compressed results for the Bland-Altman plots comparing the highest local 10gSAR predicted by Duke to that predicted by MIDA (with and without warping) at maximum 5% overestimation for 5,000 random B_1_^+^ shim sets. (A) MIDA – Duke; (B) MIKE_LIN_ – Duke; (C) MIKE_NONLIN_ – Duke; (G) MIDA – Ella; (H) MILLA_LIN_ – Ella; (I) MILLA_NONLIN_ – Ella. Also shown are histograms of maximum 10gSAR difference between (D) MIDA – Duke; (E) MIKE_LIN_ – Duke; (F) MIKE_NONLIN_ – Duke; (J) MIDA – Ella; (K) MILLA_LIN_ – Ella; (L) MILLA_NONLIN_ – Ella. See Figure 7 in the main manuscript for the uncompressed results for the Bland-Altman plots. |

# S6. Effect of linear and non-linear registration

The effect of linear registration and non-linear registration is compared in Supporting Figure S6. The largest (signed) differences in maximum 10gSAR estimation between linear and non-linear registration were -32.96%, 28.53% for MIKE_LIN_ vs MIKE_NONLIN_ and -35.65%, 42.01% in the case of MILLA_LIN_ vs MILLA_NONLIN_, respectively.

|  |
| --- |
| Supporting Figure S6: Effects of linear registration compared to non-linear registration. (A) Bland-Altman plot comparing the hottest local SAR predicted by affine linear registration of MIDA into Duke (MIKE_LIN_) versus that predicted by non-linear registration (MIKE_NONLIN_) for 5,000 B_1_^+^ shim sets. (B) Histogram of maximum 10gSAR differences between linear registration of MIDA to Duke versus non-linear registration. (C) and (D) show the corresponding plots for affine linear registration of MIDA into Ella (MILLA_LIN_) versus that predicted by non-linear registration (MILLA_NONLIN_). |

# S7. Simulated scattering matrix (S-parameters)

The simulated scattering matrices for the 8-channel coil are shown in Supporting Figure S7 for the seven different models.

|  |
| --- |
| Supporting Figure S7: Simulated S-parameters in dB; (A) MIDA model; (B) Duke model; (C) MIKE_LIN_ model; (D) MIKE_NONLIN_ model; (E) Ella model; (F) MILLA_LIN_ model; (G) MILLA_NONLIN_ model. |

**References:**

1. Christ A, Kainz W, Hahn EG, et al. The Virtual Family—development of surface-based anatomical models of two adults and two children for dosimetric simulations. *Phys Med Biol*. 2010;55(2):N23-N38. doi:10.1088/0031-9155/55/2/N01

2. Gosselin M-C, Neufeld E, Moser H, et al. Development of a new generation of high-resolution anatomical models for medical device evaluation: the Virtual Population 3.0. *Phys Med Biol*. 2014;59(18):5287-5303. doi:10.1088/0031-9155/59/18/5287

3. IT’IS Foundation. FAQ Virtual Population. https://itis.swiss/virtual-population/virtual-population/overview/faq/#04.

4. ITIS Foundation. https://www.itis.ethz.ch/virtual-population/tissue-properties/database/density/.
